# Indoxyl sulphate‐initiated activation of cardiac fibroblasts is modulated by aryl hydrocarbon receptor and nuclear factor‐erythroid‐2‐related factor 2

**DOI:** 10.1111/jcmm.18192

**Published:** 2024-03-20

**Authors:** Chiara Barisione, Daniela Verzola, Silvano Garibaldi, Paola Altieri, Anna Lisa Furfaro, Mariapaola Nitti, Giovanni Pratesi, Domenico Palombo, Pietro Ameri

**Affiliations:** ^1^ Department of Surgical and Integrated Diagnostic Sciences University of Genova Genova Italy; ^2^ Cardiac, Thoracic and Vascular Department IRCCS Ospedale Policlinico San Martino Genova Italy; ^3^ Department of Internal Medicine University of Genova Genova Italy; ^4^ Department of Experimental Medicine University of Genova Genova Italy

**Keywords:** AhR, cardiac, cardiomyocyte, fibroblast, fibrosis, indoxyl sulphate, inhibition, NRF2, renin‐angiotensin, TLR4

## Abstract

In the last decade, extensive attention has been paid to the uremic toxin indoxyl sulphate (IS) as an inducer of cardiac fibroblast (cFib) activation and cardiac fibrosis in chronic kidney disease. At cellular level, IS engages aryl hydrocarbon receptor (AhR) and regulates many biological functions. We analysed how AhR inhibition by CH‐223191 (CH) and overexpression of non‐functional (dominant negative, DN) nuclear factor‐erythroid‐2‐related factor 2 (NRF2), a transcription factor recruited by AhR, modulate the response of neonatal mouse (nm) cFib to IS. We also evaluated nm‐cardiomyocytes after incubation with the conditioned medium (CM) of IS±CH‐treated nm‐cFib. IS induced activation, collagen synthesis, TLR4 and–downstream–MCP‐1, and the genes encoding angiotensinogen, angiotensin‐converting enzyme, angiotensin type 1 receptor (*AT1r*) and neprilysin (*Nepr*) in nm‐cFib. CH antagonized IS‐initiated nm‐cFib activation, but did not affect or even magnified the other features. IS promoted NRF2 nuclear translocation and expression the NRF2 target *Nqo1*. Both pre‐incubation with CH and transfection of DN‐NRF2 resulted in loss of NRF2 nuclear localization. Moreover, DN‐NRF2 overexpression led to greater TLR4 and MCP‐1 levels following exposure to IS. The CM of IS‐primed nm‐cFib and to a larger extent the CM of IS+CH‐treated nm‐cFib upregulated *AT1r*, *Nepr* and TNFα and myostatin genes in nm‐cardiomyocytes. Hence, IS triggers pro‐inflammatory activation of nm‐cFib partly via AhR, and AhR‐NRF2 counteract it. Strategies other than AhR inhibition are needed to target IS detrimental actions on cardiac cells.

## 1 INTRODUCTION

Patients with chronic kidney disease (CKD) and end‐stage renal disease (ESRD) are at 5–10 fold higher the risk of cardiovascular disease (CVD) and cardiovascular (CV) events than age‐matched controls. Different types of CVD, including left ventricular hypertrophy (LVH) and heart failure (HF), are associated with CKD and are initiated by general and CKD‐specific factors.^1^ Cardiac fibrosis, defined as the presence of interstitial and perivascular accumulation of extracellular matrix (ECM) within the myocardium, is a major substrate and driver of LVH and HF in CKD and ESRD.^2^

Cardiac fibroblasts (cFibs) are the predominant cell type involved in cardiac fibrosis. They are quiescent in the healthy heart and become activated under pathological conditions, transdifferentiating into myofibroblasts, characterized by a fibroblast‐smooth muscle cell phenotype, α‐smooth muscle actin (αSMA) expression, contractile ability, and enhanced secretion of collagens and other ECM components, such as periostin (Postn).

Upon cardiac injury, cFib enter a proliferative phase, which amplifies their number through the upregulation of pro‐inflammatory and proliferative factors. During the subsequent ‘maturation phase’, proliferative signals decline and cFib produce and strengthen the ECM up to forming a scar to replace cardiac cells that have died.^3^, ^4^

This two‐step process of fibrosis is regulated by low‐inflammatory, T helper 2 (Th‐2) cytokines with pro‐fibrotic properties; accordingly, preclinical studies demonstrated that their selective deletion, as in the case of interleukin (IL)‐4, reduces myocardial fibrosis.^5^, ^6^

On the other side, persistent inflammation following cardiac injury can promote tissue destruction. In this context, a primary role is played by endogenous damage‐associated molecular patterns (DAMP) and overactivation of toll‐like receptor (TLR) signalling. In systemic sclerosis, tissue damage has been shown to amplify fibrosis through the TLR4/myeloid differentiation factor 2 (MD2) complex and its endogenous DAMP ligands, as demonstrated by augmented MD2 and TLR4 expression in skin biopsies from patients and by reduction of fibrosis upon MD2/TLR4 blockade in the animal model.^7^

In the last decade, extensive attention has been paid to the uremic toxin indoxyl sulphate (IS) as an inducer of cFib activation and cardiac fibrosis in CKD.^8^, ^9^, ^10^

In the cell, IS binds to aryl hydrocarbon receptor (AhR), a ligand‐activated transcription factor involved in the regulation of many biological functions under basal conditions and during stress responses to dioxin‐like compound, exogenous pollutants and endogenous toxins.^11^, ^12^, ^13^

IS may elicit redox, inflammatory, and catabolic signals, depending on the concentration and the target cell. To name a few, IS induces angiotensinogen (Agtn) in proximal tubular cells, via CREB, NF‐κB, and NADPH oxidase pathway,^14^ and upregulates tumour necrosis factor α (TNF‐α) upon AhR, NF‐κB and SOCS2 crosstalk in human macrophages.^15^ Moreover, it induces metabolic alterations (upregulation of glycolysis and pentose phosphate pathway acceleration as antioxidant stress response), via nuclear factor‐erythroid‐2‐related factor 2 (NRF2),^16^ which in turn transactivates AhR in a reciprocal crosstalk.

Previous studies proposed AhR inhibition has as therapeutic approach against IS‐induced cardiorenal damage in CKD. Promising results have been obtained with resveratrol, which also acts as AhR antagonist, both in vivo and in vitro,^17^, ^18^ as well as with selective AhR inhibitors such as α‐naphthoflavone, CH‐223191 (CH)^19^, ^20^ and geldanamycin.^21^

Here, we first analysed the profibrotic and proinflammatory effect of IS alone or in combination with CH on neonatal mouse cFib (nm‐cFib). Next, we overexpressed a non‐functional NRF2, to study the interplay between AhR and NRF2 in modulating the TLR4 inflammatory program. Finally, we evaluated the paracrine effects of IS‐primed nm‐cFib on neonatal mouse cardiomyocyte (nm‐cardiomyocyte) phenotype.

## 2 MATERIALS AND METHODS

### 2.1 Cell cultures

Isolation of cFib and cardiomyocytes from neonatal mice was performed in compliance with specific authorization (as experimental leftover from the cell cultures used in protocol project 370, DGSAF 1865‐A). Briefly,^22^ the hearts of 2‐day old C57Bl/6 mice were enzymatically digested using a 0.125 mg/mL collagenase type II solution (Worthington Biochemicals, Lakewood, New Jersey) under constant stirring, at 37°C; the recovered cells underwent double pre‐plating in fibroblast complete medium (10% FBS, 100 U/mL of penicillin, 100 mg/mL of streptomycin and 1% L‐glutamine in Dulbecco's Modified Eagle Medium, DMEM). After the second pre‐plating, adherent cells were maintained in fibroblast complete medium; the suspended cells were instead seeded onto 1% gelatin‐coated support (Sigma‐Aldrich, St. Louis, Missouri, US) in cardiomyocyte complete medium (69% DMEM, 15% M199, 10% horse serum, 5% FBS, 100 U/mL of penicillin,100 mg/mL of streptomycin and 1% L‐glutamine, Gibco‐Thermo Fisher Scientific, Waltham, Massachusetts and Sigma‐Aldrich, St. Louis, Missouri, US) and treated as nm‐cardiomyocytes. All cells were cultured at 37°C with 5%CO_2_.

### 2.2 Experimental conditions

nm‐cFib were incubated with 50 μm IS in ultrapure H_2_O and 10 μM CH‐223191 in DMSO, alone or in combination (in the latter case, CH‐223191 was added to the medium 1 h before IS) for different exposure times–from 30 min up to 72 h, depending on the experiment. To obtain the conditioned medium (CM), cells were treated with IS with or without CH for 24 h; then, the culture medium was replaced with fresh complete medium after a gentle wash with PBS. The new medium was collected 24 h later and directly used to incubate nm‐cardiomyocytes for 24 h, or stored at −80°C.

### 2.3 ROS production

nm‐cFib were treated with IS and CH‐223191, alone or in combination, for 30 and 60 min; intracellular ROS production was assessed with CellROX® Deep Red Oxidative Stress Reagents (Life Technologies; Milan, Italy), added for 30 min, and expressed as % of fluorescence intensity versus untreated, control cells (CTR). Tert‐butyl hydroperoxide (TBHP) was used as positive control for analysis calibration (not shown). Cells were analysed on Attune flow cytometer (Thermo Fisher Scientific Inc.).

### 2.4 Cell proliferation

nm‐cFib were seeded, labelled with carboxyfluoresceinsuccinimidyl ester (CFDA‐SE; Invitrogen, Milan, Italy) the day after and incubated with IS with or without CH‐223191. The proliferation rate, based on the halving of the fluorescence peak values, was recorded after 24 or 48 h. Data were analysed with the Proliferation Wizard module of the ModFit LT 4.0 software (Verity Software House, Topsham, ME, USA) and the results expressed as proliferation index (% vs. CTR). Cells were analysed on FACS calibur (Becton Dickinson).

### 2.5 Staining of nm‐cFib

Cells were grown on chamber slides to sub‐confluence, treated with IS and/or CH‐223191 and fixed in cold methanol.

For immunocytochemistry, after 48‐h treatment, cells were fixed fixation and, after quenching endogenous peroxidase (3% vol. H_2_O_2_ in PBS), incubated with αSMA mouse monoclonal antibody (Dako Agilent Pathology Solution).^23^

For immunofluorescence, treatments lasted 4 and 5 h. Cells were incubated with NRF2 rabbit polyclonal antibody (C‐20, Santa Cruz Biotechnology) or with TLR4 mouse polyclonal antibody (NB100‐56580, Novus Biologicals, Biotechne, Milan, Italy); as secondary antibodies, goat‐anti‐rabbit Alexa Fluor 568 and goat‐anti‐mouse Alexa Fluor 488 (Invitrogen, Carlsbad, CA, USA) were used.

Picrosirius red (PSR) staining (0.1% Sirius Red F3B in saturated picric acid for 1 h and wash in acidified water) was performed after a 72 h treatment to quantify collagen deposition.

### 2.6 Staining of nm‐cardiomyocytes

After 24‐h incubation with nm‐cFib CM, cells were fixed and stained for troponin T/myostatin (Mstn) expression using the following primary antibodies: Monoclonal cardiac troponin T antibody (clone 13‐11; Thermo Fisher Scientific) and Mstn polyclonal antibody (Proteintech, LaboSpace s.r.l., Milan, Italy).

### 2.7 Image analysis

Fluorescent images were captured by using a Leica DM2000 fluorescence microscope (Leica Microsystems GmbH, Wetzlar, Germany) coupled to a CCD high resolution cooled camera, using the Leica Application Suite software (Leica Microsystems).

Immunocytochemical staining was evaluated by image analysis as previously described.^23^ For PSR staining, a total of 10 fields per well were randomly chosen and images were viewed with brightfield illumination at 40×. Image analysis was performed using the Leica Q500 MC Image Analysis System (Leica, Cambridge, UK).

### 2.8 Western blot analysis

nm‐cFib were analysed in cold buffer (20 mM HEPES, 150 mM NaCl, 10% [v/v] glycerol, 0.5% [v/v] NP‐40, 1 mM EDTA, 2.5 mM DTT, 10 μg/L aprotinin, leupeptin, pepstatin A, 1 mM PMSF and Na_3_VO_4_) and total protein content determined with the Bicinchonic Protein assay kit (Merck Group, Vimodrone, Italy); 20 μg protein were resolved on SDS‐polyacrylamide gels and electro‐transferred to a PVDF membrane (Merck Group). To quantify shed TLR4 (sTLR4), 100 μL of conditioned media were concentrated to a final volume of 25 μL and run on SDS‐polyacrilamide gels. Blots were hybridized with primary antibodies against Postn (rabbit polyclonal 19899‐1‐AP, Proteintech, UK), TLR4 (mouse monoclonal sc‐293072, Santa Cruz Biotechnology) and NRF2 (rabbit polyclonal, C‐20, Santa Cruz Biotechnology) and incubated with corresponding horseradish peroxidase secondary antibodies (Cell Signaling Technology). Immunoblots were developed with Immobilon Western chemiluminescent HRP substrate (Merck). Band intensities were determined using Alliance system (Uvitec, Cambridge, UK).

### 2.9 mRNA extraction, cDNA synthesis and quantitative RT‐PCR

Total mRNA extraction was obtained with the QIAzol Lysis Reagent (Qiagen Sciences, Maryland, USA); integrity and quantification of each sample was determined with a NanoDrop ND‐1000 Spectrophotometer (NanoDrop Technologies Inc., Wilmington, DE, USA). cDNA synthesis was performed using iScript™ cDNA synthesis kit RT (Bio‐Rad Laboratories Inc., Hercules, California, USA), starting from 500 ng of mRNA. Primers for *Agtn*, angiotensin‐converting enzyme (*Ace*), angiotensin type 1 receptor (*At1r*), neprilysin (*Nepr*), *Tlr4*, monocyte chemoattractant protein‐1 (*Mcp1*), collagen type I (*Coll1*), *Tnfα*, *Mstn*, *Nfe2l2* (NRF2), NAD(P)H dehydrogenase [quinone] 1 (*Nqo1*), and β‐actin were obtained from Tib Molbiol Srl (Genova, Italy). The sequences are reported in Table 1. PCR amplification was carried out using the probe SYBR Master Mix solution (Eppendorf, Hamburg, Germany) on a MasterCycler RealPlex (Eppendorf) PCR system. Βeta‐actin was chosen as reference gene; gene expression was calculated with the ∆∆CT method of relative quantification and expressed as fold change vs. CTR cells.

**TABLE 1:** Primers used in this study.

Name	Species	Accession number	Forward	Reverse
Agtn (angiotensinogen)	Mouse	NM_007428.4	TgTgCTTgTCTAggTTggCg	gTggATgTATACgCggTCCC
Ace (angiotensin‐converting enzyme)	Mouse	NM_001130513.1	CTgggAATgAggACACggAG	TTgAACTTgggTTgggCACT
At1r *a* (angiotensin II type1 receptor)	Mouse	NM_177322	ACTCACAgCAACCCTCCAAg	ATCACCACCAAgCTgTTTCC
Nepr (neprilysin)	Mouse	NM_001289463	gAAATTCAgCCAAAgCAAgC	TCggCCTgAggAATAAAATg
Tlr4 (toll‐like receptor 4)	Mouse	NM_021297	TCAgCAAAgTCCCTgATgACA	AgAggTggTgTAAgCCATgC
Mcp‐1 (monocyte chemoattractant protein 1)	Mouse	NM_011333.3	ACCACAgTCCATgCCATCAC	TTgAggTggTTgTggAAAAg
Coll‐1 (collagen‐1)	Mouse	NM_007742.4	TCCTgCTggTgAgAAAggAT	TCCAgCAATACCCTgAggTC
Tnf‐α (tumour necrosis factor‐α)	Mouse	NM_013693	TgTAgCCCACgTCgTAgCAA	ATAgCAAATCggCTgACggT
Mstn (myostatin)	Mouse	NM_010834	TggCTCCTACTggACCTCTC	AAgATgCAgCAgTCACTCCC
NRF2 (nuclear factor‐erythroid‐2‐related factor 2)	Mouse	NM_010902	CCTCTgCTgCAAgTAgCCTC	gCTCATAgTCCTTCTgTCgCT
NQO‐1NAD(P)H dehydrogenase [quinone] 1	Mouse	NM_008706	CgACAACggTCCTTTCCAgA	gAgCAATTCCCTTCTgCCCT
β‐actin	Mouse	NM_007393.1	TTCTACAATgAgCTgCgTgTg	ggggTgTTgAAggTCTCAAA

### 2.10 Transfection with NRF2‐ dominant negative

nm‐cFib cells were transiently transfected with p_max_FPTM‐Green‐C empty vector (p_max_ EV) or p_max_FPTM‐Green‐C containing the dominant negative NRF2 protein (p_max_ NRF2‐DN), kindly provided by Prof. M. Ciriolo^24^ by using Lipofectamine 2000 (Invitrogen by Thermo Fischer, Monza, Italy) as previously described.^25^ Transfection efficiency was checked after 24 h by fluorescence microscopy. After transfection, cells were exposed for further 4 h to IS 50 μM. The efficacy of NRF2‐DN over‐epression in inhibiting NRF2‐dependent pathways was confirmed by measuring the mRNA levels of target the NRF2 target gene *Nqo1*.

### 2.11 Statistical analysis

In vitro experiments were performed at least 3 times. Data are expressed as mean ± SEM and were compared by Student's *t*‐test. Statistical significance was set at *p* < 0.05. All statistical analyses were performed using GraphPad Prism version 5.00 for Windows (GraphPad Software, San Diego, California, USA).

## 3 RESULTS

### 3.1 IS induces myofibroblast commitment of nm‐cFib in an AhR‐dependent manner

To test the effects of IS on nm‐cFib and the involvement of the AhR‐dependent pathway, nm‐cFib were incubated with 50 μM IS, with or without pre‐treatment with the AhR inhibitor CH.

**FIGURE 1:**
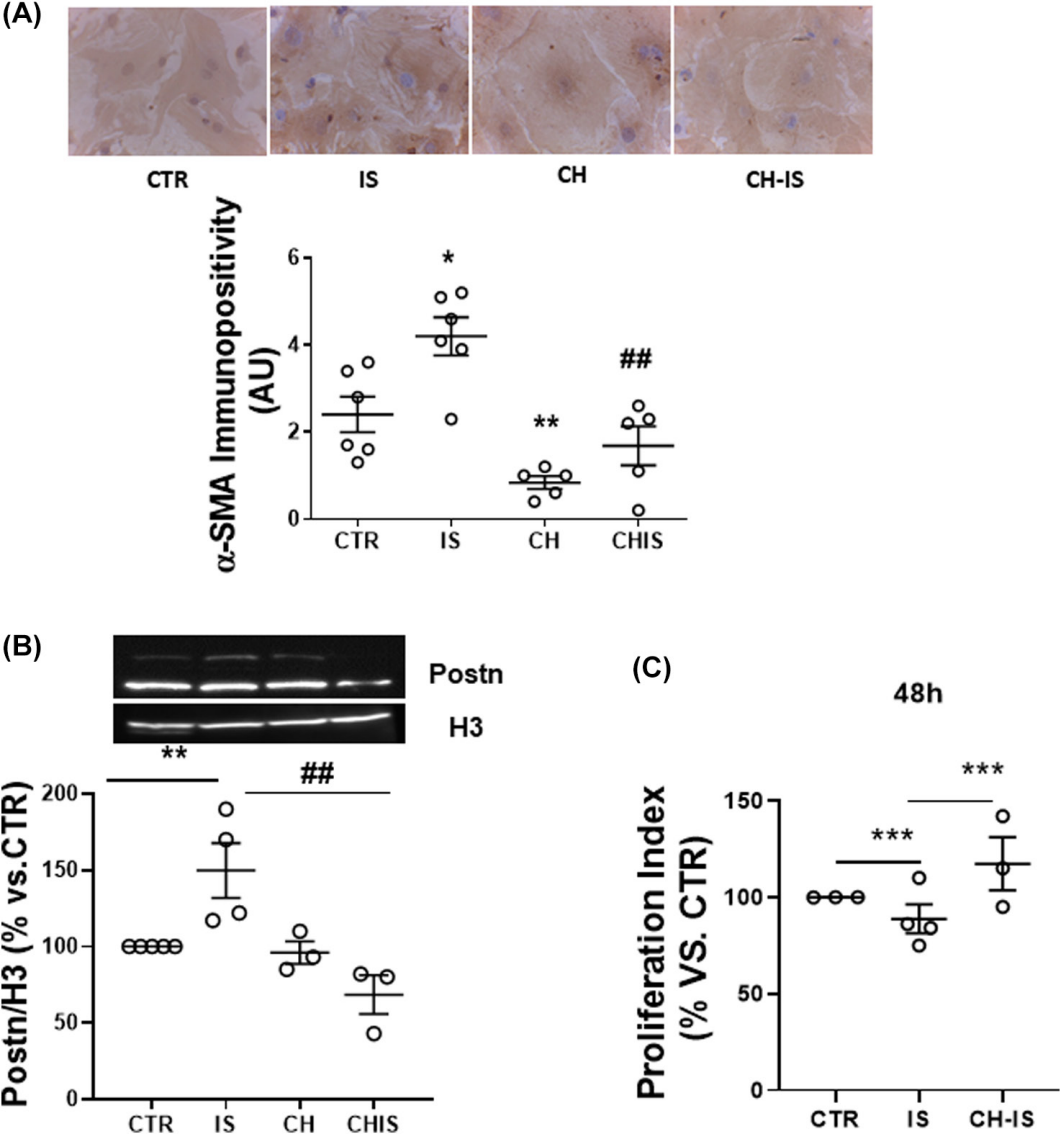
Effects of IS with or without the AhR inhibitor CH‐223191 on nm‐cFib activation. (A) Representative pictures of αSMA immunostaining (magnification 40×) and quantification of immunopositivity after a 72 h treatment; *p < 0.005 and **p < 0.01 vs. CTR, ##*p* < 0.01 versus IS. *N* = 4–5. (B) Periostin (Postn) expression after a 48 h treatment. Cell lysates were analysed by western blot, calculating the expression of the two isoforms (83 and 90 kDa); results are normalized for the histonic protein H3 and expressed as % ± SEM in respect to untreated cells (CTR) (**p < 0.01vs. CTR; ^##^ *p* < 0.01 vs. IS. *N* = 3–5. (C) Flow cytometry analysis of the proliferation index, after 48 h treatment, as detected by the probe CFSE‐DA; ***p < 0.001 of IS versus both CTR and CH‐IS. *N* = 3–4.

After 72 h‐exposure to IS, αSMA and Postn immunopositivity increased; pre‐treatment with CH alone reduced basal αSMA, but not Postn expression, and prevented the increase in these markers of activation of cFib into myofibroblasts (Figure 1A,B).

After a transient increase within 24 h (Figure S1), IS lowered the cell doubling time to 88.8 ± 3.9% of CTR. Pre‐treatment with CH enhanced proliferation of IS‐treated cells above CTR by 18 ± 5% (Figure 1C).

Thus, IS promoted the expression of myofibroblast markers and the proliferation of nm‐cFib in AhR‐dependent manner.

### 3.2 IS upregulates the renin‐angiotensin system in nm‐cFib in an AhR‐independent manner

Considering the role played by the local activation of the renin‐angiotensin system (RAS) in myocardial fibrosis,^26^ we then analysed the early (4 h) mRNA expression of *Agtn*, *Ace* and *At1r* in cells exposed to IS and pretreated with vehicle or CH.

**FIGURE 2:**
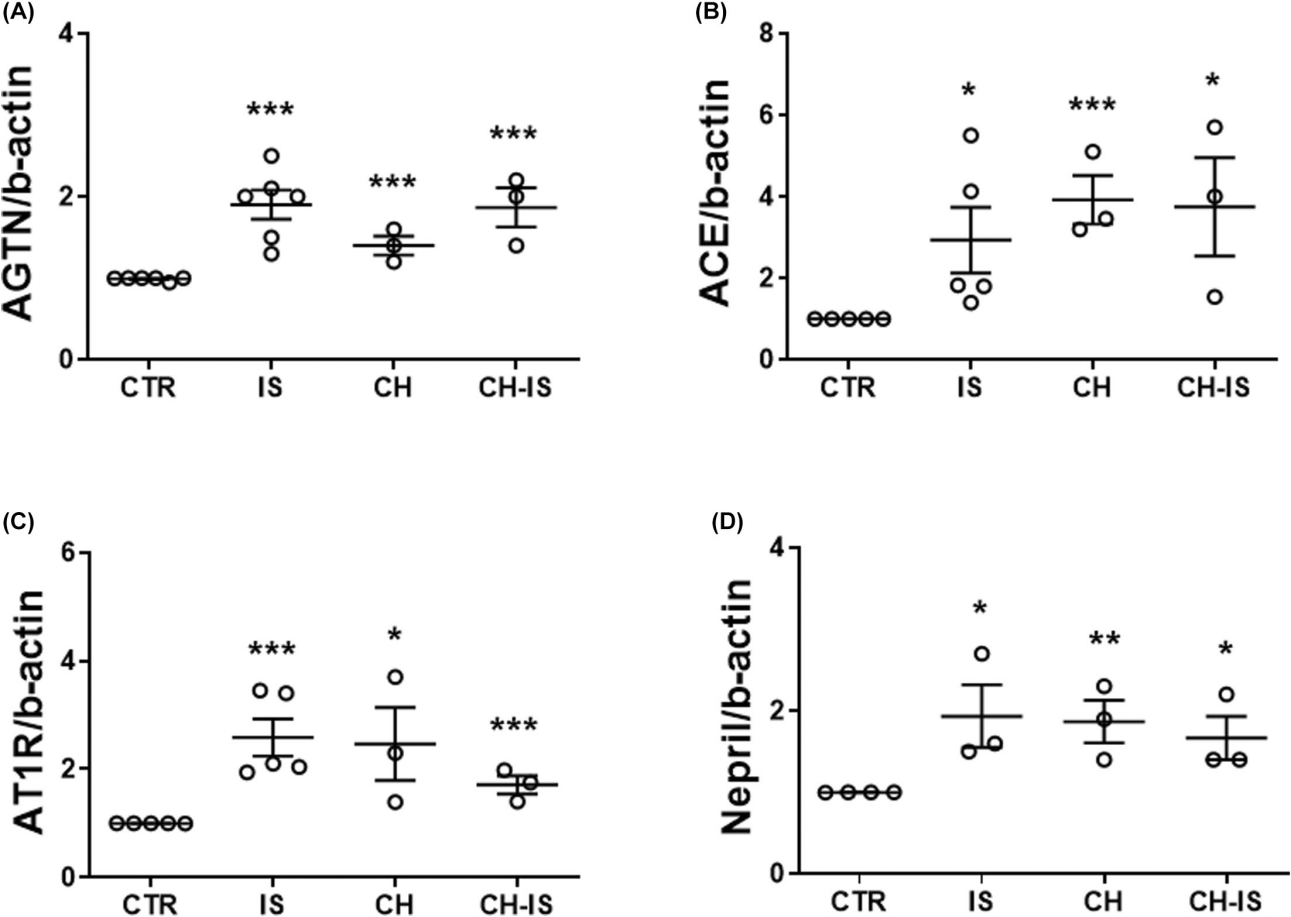
Effects of 4‐h exposure to IS with or without the AhR inhibitor CH‐223191 on the gene expression of components of the RAS in nm‐cFib. (A) Angiotensinogen (Agtn); (B) angiotensin‐converting enzyme (Ace); (C) angiotensin type 1 receptor‐(At1r); (D) neprilysin (Nepr). Data are normalized with β‐actin and plotted as fold increase ± SEM versus untreated cells (CTR); *p < 0.05; **p < 0.01; ***p < 0.001). *N* = 3–6 (A), 3–5 (B, C), and 3–4 (D).

Four‐hour incubation with IS significantly augmented the mRNA levels of *Agtn* (1.9 ± 0.2 folds), *Ace* (2.9 ± 0.8 folds) and *At1r* (1.7 ± 0.16 folds) when compared to control cells, and CH did not prevent these changes (Figure 2). Interestingly, CH per se also upregulated these genes. Furthermore, a similar trend was observed for *Nepr*, which encodes a protease modulating the RAS by reducing the levels of RAS‐counteracting natriuretic peptides. 0.7 ± 0.23 increase with IS; 1 ± 0.2 with CH; and 0.7 ± 0.2 with IS together with CH (Figure 2).

These results suggest that AhR limits the basal expression of key components of the RAS, but does not play a role in IS‐induced RAS up‐regulation.

### 3.3 IS upregulates pro‐inflammatory markers in nm‐cFib in an AhR‐independent manner

Since the RAS acts in a mutual feed‐forward loop with TLR4,^27^, ^28^ which has been reported to be under the control of AhR,^29^ we assessed TLR4 expression and the downstream pro‐inflammatory marker MCP‐1 in nm‐cFib after incubation with IS and pre‐treatment with vehicle or CH.

**FIGURE 3:**
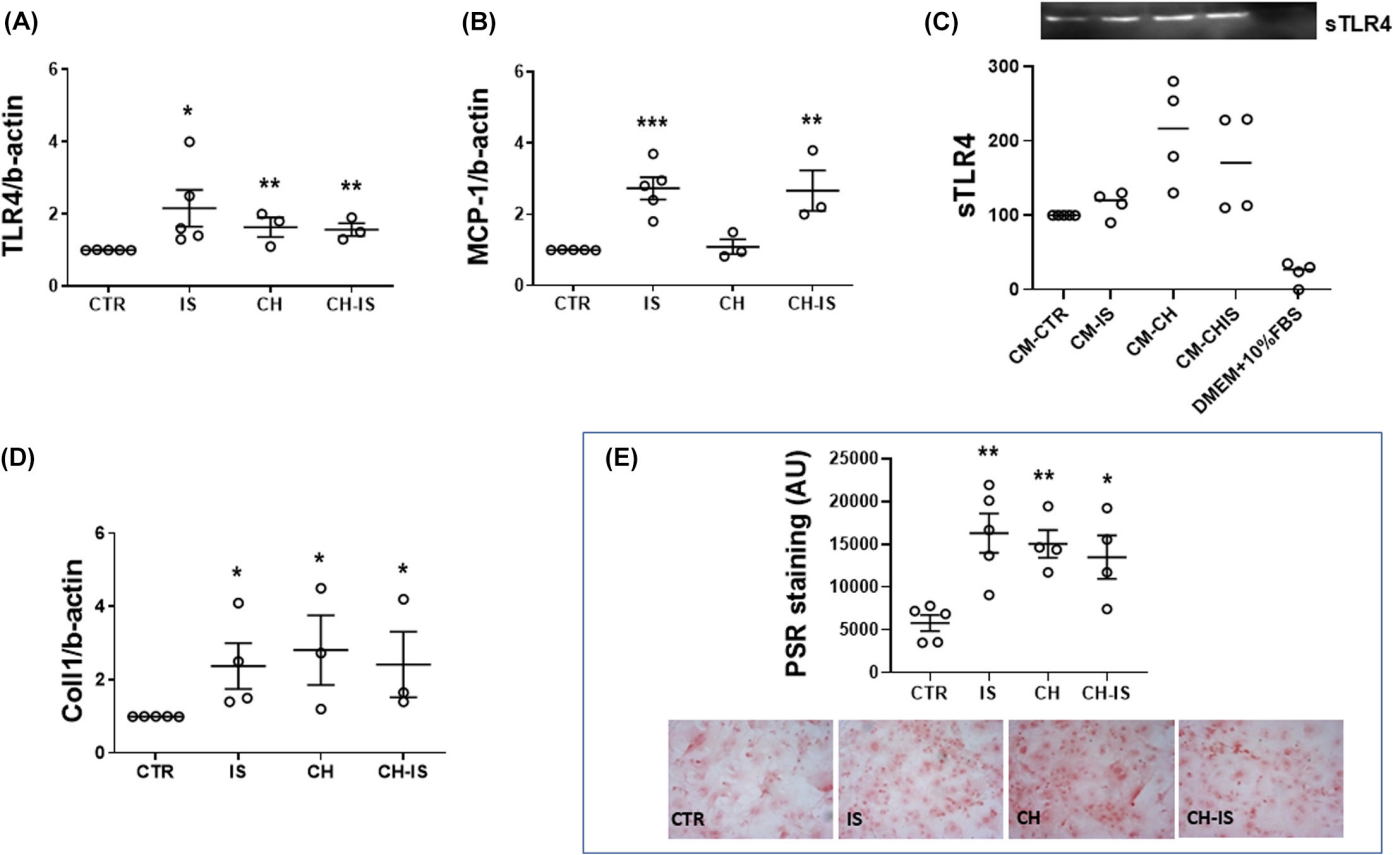
Effects of IS with or without the AhR inhibitor CH‐223191 on the expression of proinflammatory mediators and collagen deposition in nm‐cFib. (A, B) TLR4 and MCP1 mRNA after a 4 h treatment; data are normalized with β‐actin and plotted as fold increase ± SEM versus untreated cells (CTR) (*p < 0.05; **p < 0.01; ***p < 0.001). *N* = 3–5. (C) Protein expression of shed TLR4 in culture media of nm‐cFib treated for 24 h with IS and CH, alone or in combination; data, obtained by western blot analysis, are expressed as % versus media obtained from untreated cells (CM‐CTR) (*p < 0.05); as internal control, an additional sample of culture medium (DMEM + FBS) has been added. *N* = 3–5. (D) Collagen1 mRNA after a 4 h treatment; data are normalized with β‐actin and plotted as fold increase ± SEM versus untreated cells (CTR) (*p < 0.05). *N* = 3–5. (E) Collagen deposition after a 72 h treatment, as revealed by Picrosirius Red (PSR) staining; bars represents the quantification of red intensity under brightfield illumination (AU arbitrary unit; **p < 0.01; ***p < 0.001); below, representative images (magnification 10×). *N* = 4–5.

IS induced a 1‐fold increase of *Tlr4* mRNA after 4 h; both CH and the combined treatment CH plus IS also induced a modest, but significant increase of *Tlr4* mRNA (Figure 3A).

*Mcp1* mRNA expression was increased by IS, alone or in combination with CH (1.8‐ and 1.65‐ folds change, respectively); while CH alone did not affect *Mcp1* expression (Figure 3B).

Consistently with the gene expression, TLR4 protein secretion in the culture medium over 24 h was stimulated by IS, CH and CH + IS (Figure 3C).

Coll1 expression in fibroblasts has been reported to be controlled by TLR4.^7^ Consistently, 4‐h incubation with IS, CH, or CH + IS increased the expression of *Coll1* by 1–1.5 folds versus CTR, and 72‐h treatment proportionally increased collagen deposition, as revealed by PSR staining (Figure 3D,E).

Thus, IS induces the TLR4/MCP‐1 axis and collagen synthesis in nm‐cFib, in agreement with the effects on the markers of activation to myofibroblasts. Unlike the expression of αSMA and POSTN; however, modulation of TLR4, MCP‐1 and Coll1 is not blunted by inhibition of AhR.

### 3.4 NRF2 is activated by IS and limits TLR4 expression

Since AhR and NRF2 activation are mutually related, we also assessed the effects of IS on NRF2 expression and subcellular localization, as well as on reactive oxygen species (ROS) generation.

**FIGURE 4:**
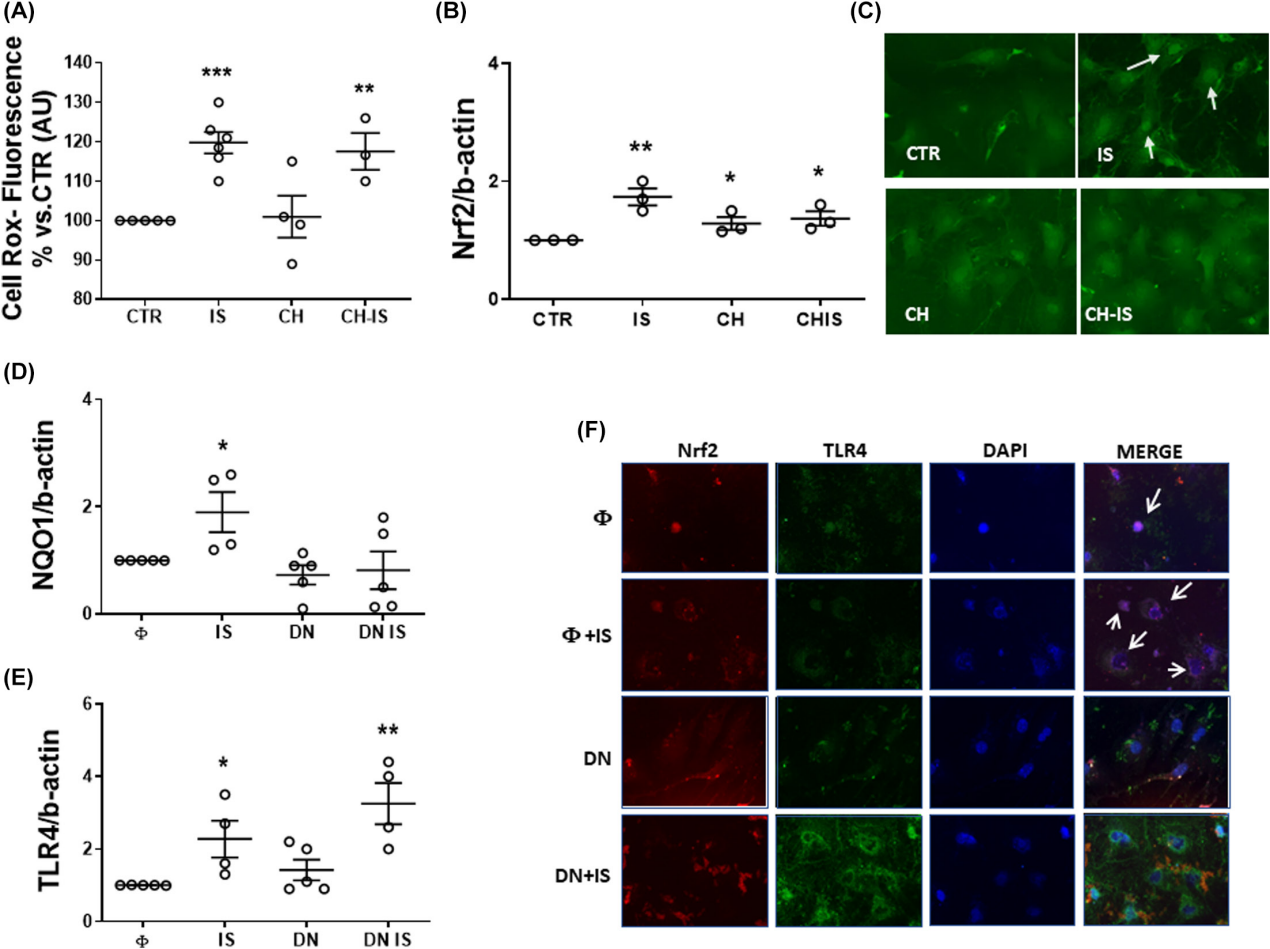
Effects of the AhR inhibitor CH‐223191 and NRF2 loss‐of‐function on NRF2 downstream signalling in nm‐cFib treated with IS. (A) Flow cytometry analysis of the ROS production, after a 30 min treatment, as detected by the probe Cell ROX; ***p < 0.001, **p < 0.01. *N* = 3–6. (B) NRF2 mRNA after a 4 h treatment; data are normalized with β‐actin and plotted as fold increase ± SEM versus untreated (CTR) cells (*p < 0.05 and **p < 0.01). *N* = 3. (C) NRF2 Intracellular distribution, detected by immunofluorescence, after a 6 h treatment. White arrows indicate nuclear localization. (D, E) NQO1 and TLR4 mRNA after a 4 h IS treatment in nm‐cFib transfected with vector alone (“empty” Φ), and with NRF2‐ dominant negative (DN‐NRF2); data are normalized with β‐actin and plotted as fold increase ± SEM versus empty (Φ) cells (*p < 0.05 and **p < 0.01). *N* = 4–5. (F) Intracellular distribution of NRF2 (red) and TLR4 (green) after a 5 h treatment with IS. White arrows indicate a NRF2 nuclear localization (purple; 40×).

IS induced a transient increase of ROS production (19.75 ± 3.05%) within 30 min. CH did not affect basal or IS‐stimulated ROS production (Figure 4A). No changes versus CTR were detectable after 1 h of incubation (Figure S2).

NRF2 mRNA was increased by 4‐h exposure to IS (0.7 ± 0.14 upregulation), CH (0.3 ± 0.1 upregulation), or CH + IS (0.4 ± 0.1 upregulation) (Figure 4B). After a 6‐h treatment, the NRF2 immunofluorescent signal was higher in the nucleus and lower in the cytoplasm of IS‐treated cells only. Cytosolic localization of NRF2 was restored pre‐treatment with CH. No significant differences of distribution were found in cells stimulated with CH alone (Figure 4C).

In cells were transfected with NRF2 dominant negative (DN‐NRF2), 4‐h treatment with IS did not modify mRNA expression of Nqo1, a downstream gene of NRF2, which was instead increased in nm‐cFib transfected with the empty vector (named ‘empty’, Φ, 0.9 ± 0.4 folds increase), further confirming that IS activated the NRF2 pathway (Figure 4D).

Moreover, in response to IS, TLR4 mRNA expression increased more in DN‐NRF2 transfected cells than in wild type ones (Figure 4E). Immunofluorescence showed that transfection with DN‐NRF2 restrained NRF2 in the cytoplasm and increased TLR4, which was further upregulated upon IS stimulation, compared to the effect observed in cells with empty vector (Figure 4F).

Thus, IS‐dependent NRF2 activation limits the effects of IS on TLR4 expression and shedding in nm‐cFib.

### 3.5 NRF2 blunts the induction of the RAS by IS

**FIGURE 5:**
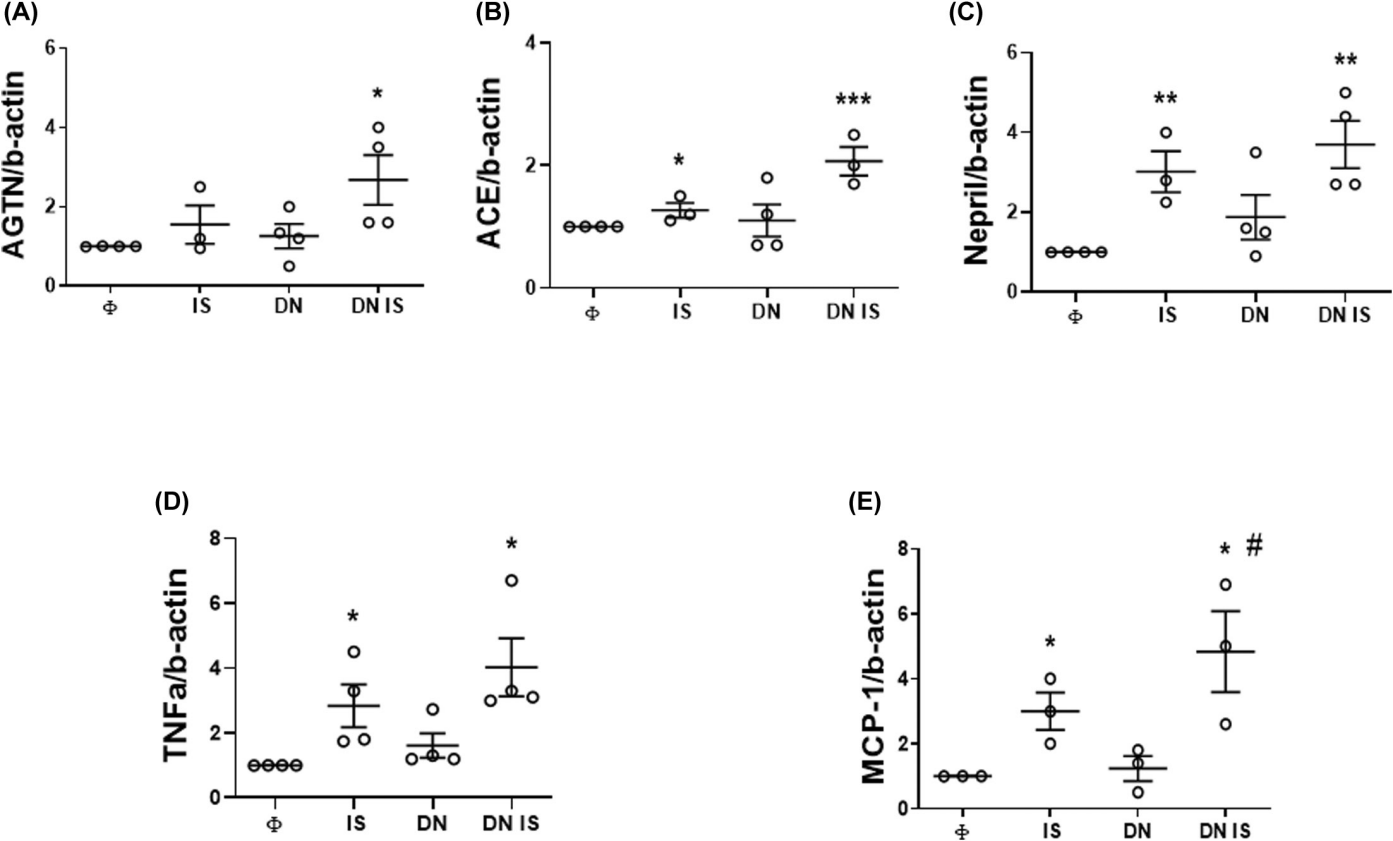
Effects of IS upon NRF2 loss‐of‐function in nm‐cFib. (A–E) Gene expression of components of the Renin‐Angiotensin system (angiotensinogen‐Agtn, angiotensin‐converting enzyme‐ACE, neprilysin‐Nepr) and inflammatory mediators (MCP‐1and TNFa) after a 4 h treatment with IS; data are normalized with β‐actin and plotted as fold increase ±SEM versus empty (Φ) cells (*p < 0.05; **p < 0.01, ***p < 0.001 vs. Φ; ^#^ *p* < 0.05 vs. Φ IS). *N* = 3–4. Φ: Cells transfected with vector alone (“empty”); DN: Cells transfected with NRF2‐ dominant negative (DN‐NRF2).

Four‐hour treatment with IS significantly increased the levels of *Ace* (0.4 ± 0.08 folds change), *Nepr* (2 ± 0.4 folds change), *Mcp1* (2 ± 0.6 folds change) and *Tnfα* (1.9 ± 1.1 folds change) in nm‐cFib transfected with empty vector in comparison with CTR (Φ). These differences were amplified in cells transfected with DN‐NRF2 and treated with IS in comparison to control cells (Φ) (*Ace*: 1 ± 0.3 folds change; *Nepr*: 2.9 ± 0.8 folds change; *Mcp1*: 3.8 ± 1.2 folds change; *Tnfα*: 2.9 ± 0.6 and 3 ± 0.9 folds increase respectively), with a significant upregulation also of *Agtn* (1.5 ± 0.7 folds change) (Figure 5A–E).

Thus, NRF2 activation limits the induction of the RAS and MCP‐1 due to IS exposure.

### 3.6 nm‐cFib similarly secrete paracrine factors that act on nm‐cardiomyocytes following IS treatment and AhR inhibition

To test the effect of IS‐induced cFib activation on nm‐cardiomyocytes, the latter were incubated with the CM of untreated (CM‐CTR), IS‐treated (CM‐IS), CH‐treated (CM‐CH), or IS+CH‐treated (CM‐CH + IS) nm‐cFib.

**FIGURE 6:**
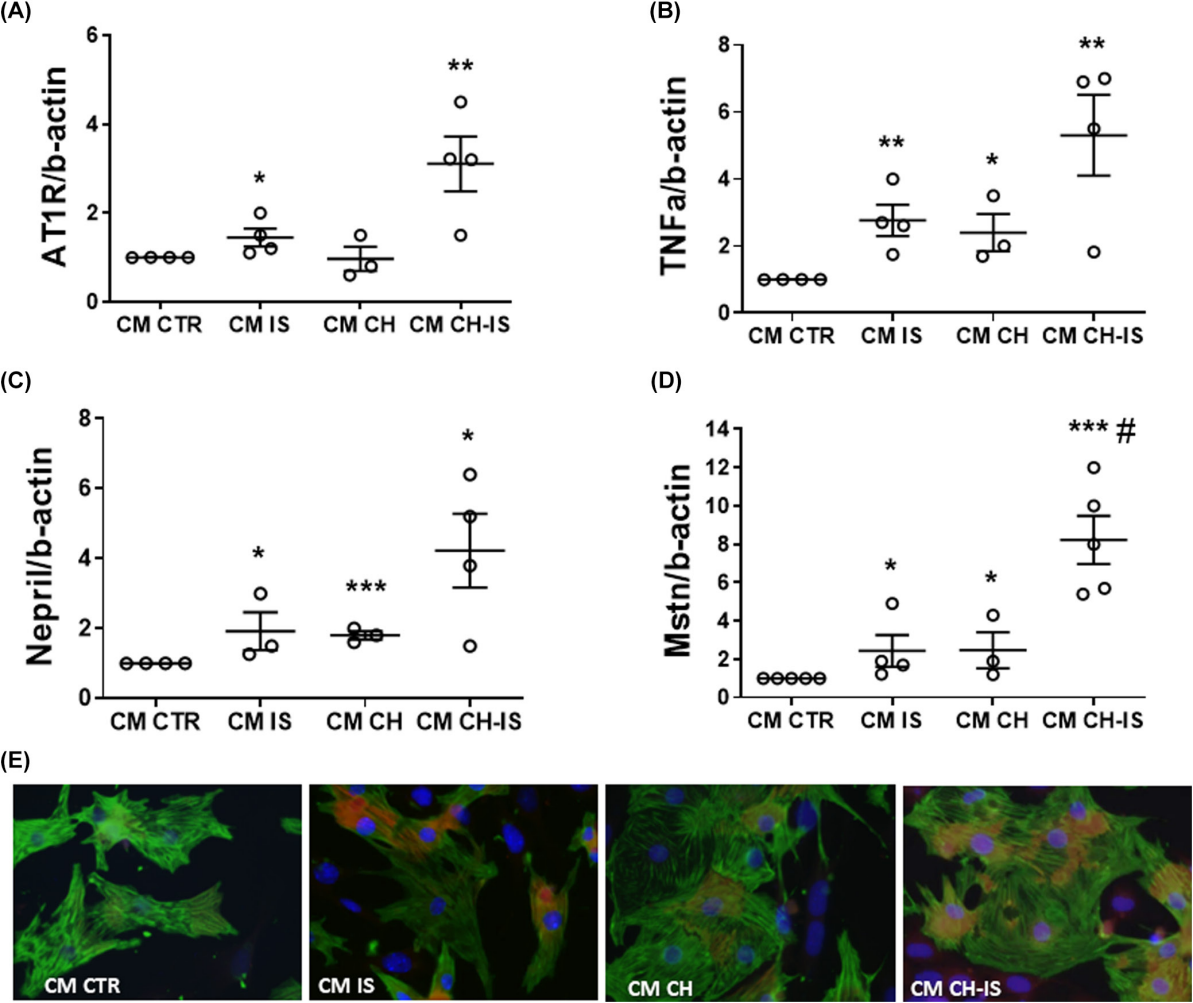
Paracrine effects of 24‐h incubation of nm‐cardiomyocytes with the conditioned medium of nm‐cFib untreated (CM CTR) or treated with IS (CM IS), CH (CM CH), or CH + IS (CM CH‐IS). (A–D) Tnfa, AT1r, Nepr, Mstn mRNA in neonatal mouse cardiomyocytes; data are normalized with β‐actin RNA and plotted as fold increase versus neonatal mouse cardiomyocytes treated with CM‐CTR (*p < 0.05, **p < 0.01, ***p < 0.001 vs. CM CTR; ^#^ *p* < 0.05 vs. CM IS). (E) Representative images of Mstn (red)/Troponin T (green) immunofluorescence in neonatal mouse cardiomyocytes incubated with nm‐cFib CM (magnification 100×). *N* = 3–5.

After 24‐h incubation, CM‐IS increased the mRNA of *Tnfa* (1.7 ± 0.4 folds change), *At1r* (0.45 ± 0.2 folds change) and *Nepr* (0.9 ± 0.4 folds change). Moreover, *Mstn*, a negative regulator of skeletal muscle growth that increases during HF development, was increased in nm‐cardiomyocytes exposed to CM‐IS (1.5 ± 0.7 folds change). CM‐CH induced a slight upregulation, comparable to IS, of *Tnfa*, *Nepr* and *Mstn*, while it did not affect *At1r* expression. Compared with CM‐IS, CM‐CH + IS amplified the transcription: *Tnfa* (4.3 ± 1.06 folds change), *At1r* (2.1 ± 0.6 folds change), *Nepr* (3.2 ± 1.05 folds change) and *Mstn* (7.2 ± 1.2 folds change) (Figure 6A–D).

Double immunofluorescence with troponin T, performed to exclude possible contaminating cells of other lineages from the primary culture, revealed that Mstn was mainly located in the cytoplasm of cardiomyocytes (Figure 6E).

## 4 DISCUSSION

In this work we show that the uremic toxin IS causes activation of nm‐cFib with the induction of collagen synthesis, pro‐inflammatory pathways, and cell‐specific RAS. Furthermore, IS‐treated nm‐cFib prime nm‐cardiomyocytes towards a pro‐inflammatory phenotype in a paracrine manner. We also report that AhR and NRF2 blunt part of IS effects on nm‐cFib and, secondarily, nm‐cardiomyocytes.

CKD is an established risk factors for LVH and HF via several, distinct mechanisms.^30^

In the last decade, a link between CKD and HF has been recognized in non‐haemodialyzable uremic toxins, which contribute to chronic cardiac dysfunction by acting on cardiac cells. One such toxin is IS, a product of diet‐derived tryptophan that is converted to indole by intestinal bacteria and then to IS in other organs.

Clinically, plasma concentrations of IS are associated with incident HF in ESRD patients^31^ and are a strong and independent predictor of atrial fibrillation recurrence in patients undergoing successful catheter ablation.^32^ Moreover, IS levels predict CV events in subjects with HF.^33^

We already reported that mild‐to‐moderate increases of IS (i.e. at concentrations up to 20 μM), as found during transition from mild to moderate CKD, promote monocyte differentiation towards macrophages with low‐inflammatory, pro‐fibrotic potential, through an AhR/NRF2‐HO1 signalling, sustaining chronic inflammation and maladaptive vascular remodelling.^34^ These results are somehow in contrast with the observation that high IS concentrations (250 μM), as found in patients with advanced CKD, elicited an oxidative stress‐based damage through NRF2 downregulation,^35^ but the discrepancy can be explained by the fact that the IS‐induced effects are non‐linearly dose‐dependent.

We also previously reported that IS trigger renal fibroblast activation through HSP90/Smad 2/3 downstream of AhR.^36^

We now expand these prior observations by showing that IS also activates nm‐cFib. Interestingly, IS also upregulated a cell‐intrinsic RAS in nm‐cFib, as well as the endopeptidase *Nepr*. The AhR activator dioxin was found to enhance collagen deposition and aSMA, preluding to liver fibrosis,^37^ and was implicated in the basal expression of matrix metalloproteinase 1 by fibroblasts, which is crucial in collagen remodelling.^38^ When we challenged AhR inhibition for cardioprotection in nm‐cFib exposed to IS, we found that it has diverse consequences. While pre‐treatment with CH diminished IS‐induced myofibroblast transition, it did not counteract the effects of IS on TLR4, the RAS, and collagen expression and deposition, nor the secretion of paracrine factors affecting nm‐cardiomyocytes.

Therefore, even though other authors reported the efficacy of AhR inhibition in contrasting RAS activation due to IS in CKD,^39^ our data indicate that this strategy may not be the optimal one to antagonize the actions of IS on cFib and, indirectly, on nm‐cardiomyocytes.

AhR has been already reported to control MCP‐1 and TLR4,^29^ and TLR4 to act in a mutual feed‐forward loop with the RAS.^27^, ^28^ It was also shown that, in macrophage‐like cells, lipopolysaccharide‐dependent TLR4 stimulation induced MCP‐1 expression, which could be reversed by AhR‐independent mechanisms.^40^

Notably, AhR undergoes to a wide epigenetic modulation, and AhR‐mediated effects of IS depend on the concentration, the length of stimulation and the tissue‐specific stress response.^41^, ^42^

Therefore, a deeper understanding of how and when IS engages AhR is essential to inform the development of novel anti‐inflammatory and anti‐fibrotic therapies.

In nm‐cFib, inhibition of AhR by CH unleashed the expression of genes and proteins that were also stimulated by IS. Similarly, NRF2 silencing led to enhanced effects of IS on TLR‐4, MCP‐1 and the RAS.

NRF2 has long been considered a cornerstone for cell protection and homeostasis in response to oxidative stress. However, while several studies refer the benefit of potentiated Keap1/NRF2/HO‐1 signalling pathway in suppressing oxidative/nitrative stress and inflammatory response,^43^, ^44^ others report detrimental effects of NRF2 upregulation. In a mouse model of pressure overload‐induced cardiac remodelling, the impaired integrity of myocardial autophagy turned off the NRF2‐mediated cardioprotection, and switched on NRF2‐mediated cardiac dysfunction by inducing angiotensinogen transcription, which exacerbates cardiac maladaptation.^45^

In our experimental setting, the co‐activation of AhR and NRF2 restrained inflammation in favour of profibrotic changes. These results are consistent with those obtained in monocytes, where IS evoked only a transient rise in ROS production, but sufficient to promote the differentiation towards profibrotic macrophages.^34^

Finally, the CM of IS‐treated nm‐cFib served as a proinflammatory microenvironment for nm‐cardiomyocytes and these effects were amplified when nm‐cardiomyocytes were also incubated with CH.

cFib drive cardiomyocyte survival and function in the different stage of the life, from myocardium development up to aging, providing a scaffold to prevent anoikis and mediating metabolic/inflammatory paracrine signals. On the other hand, cardiomyocytes are critical contributors to the myocardial fibrotic programs, in response to injurious stimuli (mechanical stress, metabolic dysfunction, activation of neurohormonal processes such as the renin–angiotensin–aldosterone system, or inflammatory cytokines) that may induce cFib activation.^36^, ^46^

In the present work, we demonstrate that AhR induces fibrosis and modulates TLR4 expression in IS‐treated nm‐cFib through AhR/NRF2 co‐activation, while AhR inhibition with CH‐ reduces fibrosis but hampered NRF2 activity, upregulating TLR4, the RAS and paracrine mediators of inflammation in nm‐cardiomyocytes.

In conclusion, we provide molecular hints to design multiple‐targeting pharmacological strategies of protection against IS‐induced cFib activation and intracardiac RAS expression and inflammation.

## AUTHOR CONTRIBUTIONS

**Chiara Barisione:** Conceptualization (lead); data curation (lead); formal analysis (equal); investigation (lead); methodology (lead); project administration (lead); writing – original draft (lead). **Daniela Verzola:** Conceptualization (supporting); data curation (supporting); investigation (supporting); methodology (supporting); supervision (supporting); validation (supporting); writing – review and editing (supporting). **Silvano Garibaldi:** Data curation (supporting); investigation (supporting); methodology (supporting); validation (supporting); visualization (supporting); writing – original draft (supporting). **Paola Altieri:** Formal analysis (supporting); investigation (supporting); visualization (supporting); writing – review and editing (supporting). **Anna Lisa Furfaro:** Investigation (supporting); methodology (supporting); writing – review and editing (supporting). **Mariapaola Nitti:** Conceptualization (supporting); data curation (supporting); formal analysis (supporting); investigation (lead); methodology (lead); resources (supporting); validation (supporting); writing – original draft (supporting). **Giovanni Pratesi:** Funding acquisition (supporting); project administration (supporting); resources (lead); visualization (supporting); writing – review and editing (supporting). **Domenico Palombo:** Conceptualization (supporting); project administration (supporting); resources (supporting); validation (supporting); writing – review and editing (supporting). **Pietro Ameri:** Formal analysis (lead); funding acquisition (lead); methodology (supporting); resources (lead); supervision (lead); validation (supporting); writing – review and editing (lead).

## CONFLICT OF INTEREST STATEMENT

P.A. received speaker, advisory board and consultancy fees from Boehringer Ingelheim, Astra Zeneca, Bayer, Daiichi Sankyo, Janssen, and MSD, all outside the scopes of the present work.

The other authors have no conflicts of interest to disclose.

## Supporting information


Figure S1.

## Data Availability

The data that support the findings of this study are available from the corresponding author upon reasonable request.
