# Supplementary material for: Indoxyl sulphate‐initiated activation of cardiac fibroblasts is modulated by aryl hydrocarbon receptor and nuclear factor‐erythroid‐2‐related factor 2
Source: J Cell Mol Med. 2024 Mar 20;28(7):e18192. doi: 10.1111/jcmm.18192 (PMC10951876; doi:10.1111/jcmm.18192)
Supplement: Supplementary file 1 — Figure S1. [file JCMM-28-e18192-s001.docx]

**Indoxyl sulphate-initiated activation of cardiac fibroblasts is modulated by aryl hydrocarbon receptor and nuclear factor-erythroid-2-related factor 2**

Chiara Barisione, Daniela Verzola, Silvano Garibaldi^4^, Paola Altieri, Anna Lisa Furfaro, Mariapaola Nitti, Giovanni Pratesi, Domenico Palombo, Pietro Ameri

**Supplementary Material**

**Supplementary Figure 1**

Flow cytometry analysis of the proliferation index, as detected by the probe CFSE-DA, after 24 hours of treatment IS 50 µM with or without CH 10µM. ^∗∗∗^ p < 0.001 of IS vs both CTR and CH-IS.

**Supplementary Figure 2**

Flow cytometry analysis of ROS production after 1 hour treatment of nm-cFib with IS 50 µM with or without CH 10µM; results are expressed as % ± SEM as compared to untreated cells (CTR).
